# An Analysis of Clinicopathological Outcomes and the Utility of Preoperative MRI for Patients Undergoing Resection of Mucinous and Non-Mucinous Colorectal Cancer Liver Metastases

**DOI:** 10.3389/fonc.2022.821159

**Published:** 2022-02-21

**Authors:** Ian S. Reynolds, Paul M. Cromwell, Éanna J. Ryan, Erinn McGrath, Rory Kennelly, Ronan Ryan, Niall Swan, Kieran Sheahan, Des C. Winter, Emir Hoti

**Affiliations:** ^1^ Department of Hepatobiliary and Liver Transplant Surgery, St Vincent’s University Hospital, Dublin, Ireland; ^2^ Department of Colorectal Surgery, St Vincent’s University Hospital, Dublin, Ireland; ^3^ Department of Histopathology, St Vincent’s University Hospital, Dublin, Ireland; ^4^ Department of Radiology, St Vincent’s University Hospital, Dublin, Ireland

**Keywords:** colorectal cancer, colorectal cancer liver metastasis, mucinous colorectal cancer, liver resection, surgical oncology, mucinous colorectal cancer liver metastasis

## Abstract

**Background and Aims:**

Mucinous colorectal cancer has traditionally been associated with high rates of recurrence and poor long-term survival. There is limited published data on outcomes for patients undergoing liver resection for metastatic mucinous colorectal cancer. The aim of this study was to compare the clinicopathological outcomes for patients with mucinous colorectal cancer liver metastases (CRCLM) undergoing liver resection to a matched group of patients with adenocarcinoma not otherwise specified (NOS) and to evaluate the accurary of preoperative magnetic resonance imaging (MRI) at detecting the presence of mucin in liver metastases.

**Materials and Methods:**

Patients with mucinous CRCLM undergoing liver resection were matched 1:3 to patients with adenocarcinoma NOS CRCLM. Clinicopathological data from the primary tumour and metastatic lesion were collected and compared between the groups. Hepatic recurrence-free, disease-free and overall survival were compared between the groups. The ability of preoperative MRI to detect mucin in CRCLM was also evaluated.

**Results:**

A total of 25 patients with mucinous CRCLM underwent surgery over the 12-year period and were matched to 75 patients with adenocarcinoma NOS. Clinicopathological findings were similar between the groups. Resection of mucinous CRCLM was feasible and safe with similar levels of morbidity to adenocarcinoma NOS. There were no differences identified in hepatic recurrence-free (p=0.85), disease-free (p=0.25) and overall survival (p=0.98) between the groups. MRI had a sensitivity of 31.3% in detecting the presence of mucin in CRCLM.

**Conclusion:**

Patients with mucinous CRCLM in this study had similar outcomes to patients with adenocarcinoma NOS. Based on our findings, histological subtype should not be taken into account when deciding on resectability of CRCLM.

## Introduction

Resection of colorectal cancer liver metastasis (CRCLM) has become the standard of care in patients who are physiologically suitable with potentially curable disease and is typically associated with 5-year overall survival rates of 25-40%, but as high as 58% in some series (1–3). The criteria for resection of CRCLM is continuing to expand and in certain patients with multifocal bilateral disease an attempt at curative resection might still be considered if the patient is deemed suitable for a two staged hepatectomy (TSH) (4, 5). The use of perioperative chemotherapy, targeted therapy, transarterial chemoembolisation and local ablative techniques are also part of the armamentarium of the multidisciplincary team treating patients with CRCLM (6–9). As the boundaries continue to be pushed some patients are being offered repeat resection or local ablative techniques when diagnosed with local hepatic recurrence after their first resection (10, 11). Long-term outcomes are expected to improve as further advances in surgical techniques and precision medicine enter mainstream clinical practice.

In the setting of primary colorectal cancer (CRC) there has been some focus on outcomes based on histological subtype. Adenocarcinoma not otherwise specified (NOS) is the most frequently diagnosed histological subtype of CRC followed by mucinous adenocarcinoma which accounts for approximately 10-15% of cases (12). While primary mucinous CRC has traditionally been associated with worse outcomes, more recent evidence suggests that modern treatment methods appear to have closed the prognostic gap between mucinous adenocarcinoma and adenocarcinoma NOS (13, 14). To date only a small number of studies have focused on outcomes for patients with mucinous CRCLM undergoing surgery and the results have been conflicting (15–17). Most of the studies that have compared mucinous CRCLM to adenocarcinoma NOS CRCLM have done so without any form of matching and in many cases the primary tumours were larger in size, had higher pT and pN stages and were more likely to be poorly differentiated, all factors which likely contribute to a worse prognosis (18–20). Interestingly, not all CRCLM derived from mucinous primary tumours contain mucin in the liver metastasis. Due to tumour heterogeneity a preoperative liver biopsy may fail to determine if a CRCLM is mucinous or not. There has been some discussion regarding the ability of preoperative imaging techniques such as positron emission tomography (PET) and magnetic resonance imaging (MRI) to determine whether or not a CRCLM contains mucin (21, 22). Several features on MRI such as high signal intensity on T2 weighted imaging and rim enhancement can be helpful in identifying mucinous CRCLM, however, not all cases can be identified by these features (23, 24). The relevance in determining whether a CRCLM contains mucin or not is unknown and in some cases metastatic lesions may have a mucin component which is due to preoperative treatment response.

The aim of this study was to compare the clinicopathological features, hepatic recurrence-free survival, disease-free survival and overall survival in a group of patients undergoing surgery for mucinous CRCLM matched with a group of patients undergoing surgery for adenocarcinoma NOS CRCLM. This study also sought to determine the accuracy of preoperative MRI in determining the presence of a mucinous component in CRCLM.

## Materials and Methods

A retrospective review of our institutional review board-approved prospectively maintained CRCLM database was performed and all cases of mucinous CRCLM that were resected during the 12-year period from January 1^st^ 2009 until December 31^st^ 2020 were identifed. A mucinous tumour was defined as a tumour in which more than 50% of the lesion was composed of pools of extracellular mucin (25). The definition of whether or not a tumour was mucinous was determined based on the histology of the primary tumour. Each mucinous case was matched with three adenocarcinoma NOS cases for sex, age, primary tumour sidedness, primary tumour size (mm), primary tumour stage, largest liver metastasis size (mm), the anatomical distribution of the liver metastasis, laterality (whether uni- or bi-lobar) and finally the number of liver metastases.

Clinicopathological characteristics of the primary colorectal tumour including; tumour location, mean tumour size, tumour differentiation, TNM stage, perineural invasion (PNI), lymphovascular invasion (LVI), extramural venous invasion (EMVI), tumour margin, tumour perforation, resection margin, mismatch repair (MMR) status, *KRAS* and *BRAF* mutation status as well as adjuvant treatment status were compared between the groups. Clinicopathological, operative and perioperative treatment status of the metastatic tumours were also compared between the groups. In particular; tumour differentiation, maximum dimension of the largest metastasis, number of metastases, presence of bilobar metastases, presence of vascular invasion, resection margin status, the extent of resection, modality of resection, median operating time, length of stay, operative morbidity and the use of pre and post-operative systemic therapy were compared between the groups. Median hepatic recurrence-free survival, disease-free survival and overall survival were calculated and compared between the two groups. Overall survival at 1, 3 and 5-years were also calculated for both groups.

Preoperative MRI scans were performed using gadoxetate disodium which is a hepatospecific paramagnetic gadolinium based contrast agent.The preoperative MRI scan of the liver where available was reviewed for each patient by a consultant radiologist with a special interest in hepatobiliary radiology. The radiologist was blinded as to the histology of the primary tumour and the metastasis. The radiologist documented whether or not there were radiological features of a mucin containing tumour on the MRI scan. Potential radiological features compatible with mucinous tumours included; high signal intensity on T2 weighted imaging, peripheral enhancement on vascular phase imaging and washout of contrast media on delayed phased imaging. The official histology report of the corresponding liver metastasis was then reviewed to see if it had a mucinous component. A confusion matrix was used to evaluate the performance of MRI in predicting if a tumour had a mucinous component or whether it was solid. Sensitivity, specificity, positive predictive value (PPV), negative predictive value (NPV) and overall accuracy were calculated.

A propensity score matching method was employed to address the inherent selection bias of this observational study. A propensity score was calculated for the likelihood of the primary tumour being of mucinous histology accounting for the covariates of sex, age, primary tumour sidedness, primary tumour size (mm), primary tumour stage, largest liver metastasis size (mm), the anatomical distribution of the liver metastasis, laterality (whether uni- or bi-lobar) and finally the number of liver metastases. A nearest neighbour matching technique was performed. The means and distributions of the matched samples were then compared to evaluate the quality of the match performed. Outcomes were then evaluated on the propensity matched samples, thus controlling for unbalanced covariates. Contingency tables were analysed using Fisher’s exact test. Means were compared using an unpaired *t* test. Medians were compared using the Mann-Whitney test. Median hepatic recurrence-free, disease-free and overall survival were calculated using Kaplan-Meier methods and the log-rank test. Time to last follow-up, recurrence or death were measured from the date of liver resection. Number at risk for each time point was calculated and is displayed below the Kaplan-Meier curves. A *p* value of <0.05 was deemed to be statistically significant. Statistical analysis was carried out using IBM® SPSS® Statistics 27 and GraphPad Prism version 9.2.0.

## Results

A total of 25 patients underwent resection of mucinous CRCLM during the study period and these patients were matched with a group of 75 patients who underwent liver resection for CRCLM derived from adenocarcinoma NOS. Clinicopathological variables, staging and genomic features of the primary tumours from both groups were similar [See (**Table 1**)].

**Table 1:** Primary tumour data.

	Mucinous (n=25)	Non-Mucinous (n=75)	*P* value
Mean age (+/- SEM)	60.6 (+/-2.3)	61.3 (+/-1.1)	0.76
Female gender	9/25 (36.0%)	26/75 (34.7%)	1.00
Primary in rectum	7/25 (28.0%)	18/75 (24.0%)	0.79
Right sided primary	13/25 (52.0%)	30/75 (40.0%)	0.35
Mean tumor size (+/-SEM)	50.3 (+/-6.8) mm	39.8 (+/-2.3) mm	0.07
Poorly differentiated	4/25 (16.0%)	10/75 (13.3%)	0.75
pT3/T4 tumor	20/25 (80.0%)	69/75 (92.0%)	0.14
Node positive	16/25 (64.0%)	48/75 (64.0%)	1.00
Median lymph node yield	17.5 (range 2-38)	17 (range 3-39)	0.62
Stage IV at diagnosis	13/25 (52.0%)	39/75 (52.0%)	1.00
Perineural invasion	7/25 (28.0%)	24/75 (32.0%)	0.81
Lymphovascular invasion	11/25 (44.0%)	36/75 (48.0%)	0.82
Extramural venous invasion	10/25 (40.0%)	36/75 (48.0%)	0.64
Infiltrative tumor margin	10/17 (58.8%)	52/74 (70.3%)	0.39
Tumour perforation	1/25 (4.0%)	9/75 (12.0%)	0.44
Positive resection margin	1/25 (4.3%)	2/75 (2.7%)	1.00
*KRAS*/*NRAS* mutated	12/22 (54.5%)	25/62 (40.3%)	0.32
*BRAF* mutated	2/15 (13.3%)	0/18 (0.0%)	0.20
MMR deficient	2/24 (8.3%)	1/75 (1.3%)	0.14
Adjuvant chemotherapy	18/25 (72.0%)	54/75 (72.0%)	1.00
Intra-abdominal recurrence	9/25 (36.0%)	20/75 (26.7%)	0.45

Mean age, Mean age at diagnosis of primary tumor; SEM, standard error of the mean; pT, pathological tumor stage; KRAS, Kirsten rat sarcoma viral oncogene homolog; NRAS, Neuroblastoma RAS viral oncogene homolog; BRAF, Murine sarcoma viral oncogene homolog B1; MMR, Mismatch repair.

There was no statistical differences in the proportion of patients undergoing two-staged liver resection (p=1.00), combined liver and colon or rectal resection (p=0.60) or minimally invasive liver resection (p=1.00). The positive resection margin rate was 20.0% in the adenocarcinoma NOS group and 4.0% in the mucinous group, however, this difference did not reach statistical significance (p=0.07). There was no difference in the mean number of lesions (1.88 vs 2.32, p=0.32), the presence of bilobar disease (28.0% vs 32.0%, p=0.81) or the presence of vascular invasion (12.0% vs 30.7%, p=0.07) between the two groups. Length of stay (10 days vs 9 days, p=0.97), liver specific morbidity (12.0% vs 16.0%, p=0.76) and 30-day mortality (4.0% vs 1.3%, p=0.44) were also similar between the two groups [See (**Table 2**)]. A single death occurred in the mucinous group in a patient who infarcted their liver remnant on postoperative day 4 after undergoing an extended right hepatectomy and resection of segment 1. The death in the non-mucinous group occurred due to an in-hospital intracranial haemorrhage, complicated by hospital acquired pneumonia. No difference was found in the proportion of patients undergoing preoperative systemic treatment (72.0% vs 50.7%, p=0.07) or postoperative systemic treatment (64.0% vs 77.3%, p=0.20).

**Table 2:** Liver metastases data.

	Mucinous (n=25)	Non-Mucinous (n=75)	*P* value
Mean age (+/- SEM)	61.6 (+/-2.2)	62.2 (+/-1.1)	0.82
Liver metastasis diagnosed within 6 months of primary	14/25 (56.0%)	42/75 (56.0%)	1.00
Two stage resection	2/25 (8.0%)	8/75 (10.7%)	1.00
Combined resection	5/25 (20.0%)	21/75 (28.0%)	0.60
Liver first resection	4/25 (16.0%)	7/75 (9.3%)	046
Laparoscopic resection	4/25 (16.0%)	13/75 (17.3%)	1.00
Type of resection			0.51
*Wedge resection*	1/25 (4.0%)	2/75 (2.7%)	
*Multiple wedge resections*	2/25 (8.0%)	2/75 (2.7%)	
*Segmental resection*	4/25 (16.0%)	16/75 (21.3%)	
*Multisegmental resection*	8/25 (32.0%)	33/75 (44.0%)	
*Right hepatectomy*	5/25 (20.0%)	7/75 (9.3%)	
*Right hepatectomy and RFA*	0/25 (0.0%)	1/75 (1.3%)	
*Extended right hepatectomy*	1/25 (4.0%)	2/75 (2.7%)	
*Left lateral segmentectomy*	0/25 (0.0%)	1/75 (1.3%)	
*Left lobectomy*	1/25 (4.0%)	0/75 (0.0%)	
*Left hepatectomy*	0/25 (0.0%)	3/75 (4.0%)	
*Two stage procedure*	2/25 (8.0%)	8/75 (10.7%)	
*Extended right hepatectomy and segmental resection*	1/25 (4.0%)	0/75 (0.0%)	
Median operating time (range)	195 (64-373) mins	208 (60-542) mins	0.97
Median length of stay (range)	10 (2-78) days	9 (4-47) days	0.97
30-day mortality	1/25 (4.0%)	1/75 (1.3%)	0.44
Liver specific morbidity	3/25 (12.0%)	12/75 (16.0%)	0.76
Poorly differentiated	2/25 (8.0%)	13/75 (17.3%)	0.34
Mean size of largest metastasis (+/-SEM)	45.8mm (+/-8.3)	39.6mm (+/-3.9)	0.45
Positve resection margin	1/25 (4.0%)	15/75 (20.0%)	0.07
Mean number of lesions (+/-SEM)	1.88 (+/-0.23)	2.32 (+/-0.24)	0.32
Bilobar disease	7/25 (28.0%)	24/75 (32.0%)	0.81
Vascular invasion	3/25 (12.0%)	23/75 (30.7%)	0.07
Preoperative treatment	18/25 (72.0%)	38/75 (50.7%)	0.07
Postoperative treatment	16/25 (64.0%)	58/75 (77.3%)	0.20
Repeat resection for recurrence	6/25 (24.0%)	8/75 (10.7%)	0.11

Mean age, Mean age at diagnosis of liver metastasis; SEM, standard error of the mean; RFA, radiofrequency ablation.

**Figure 1:**
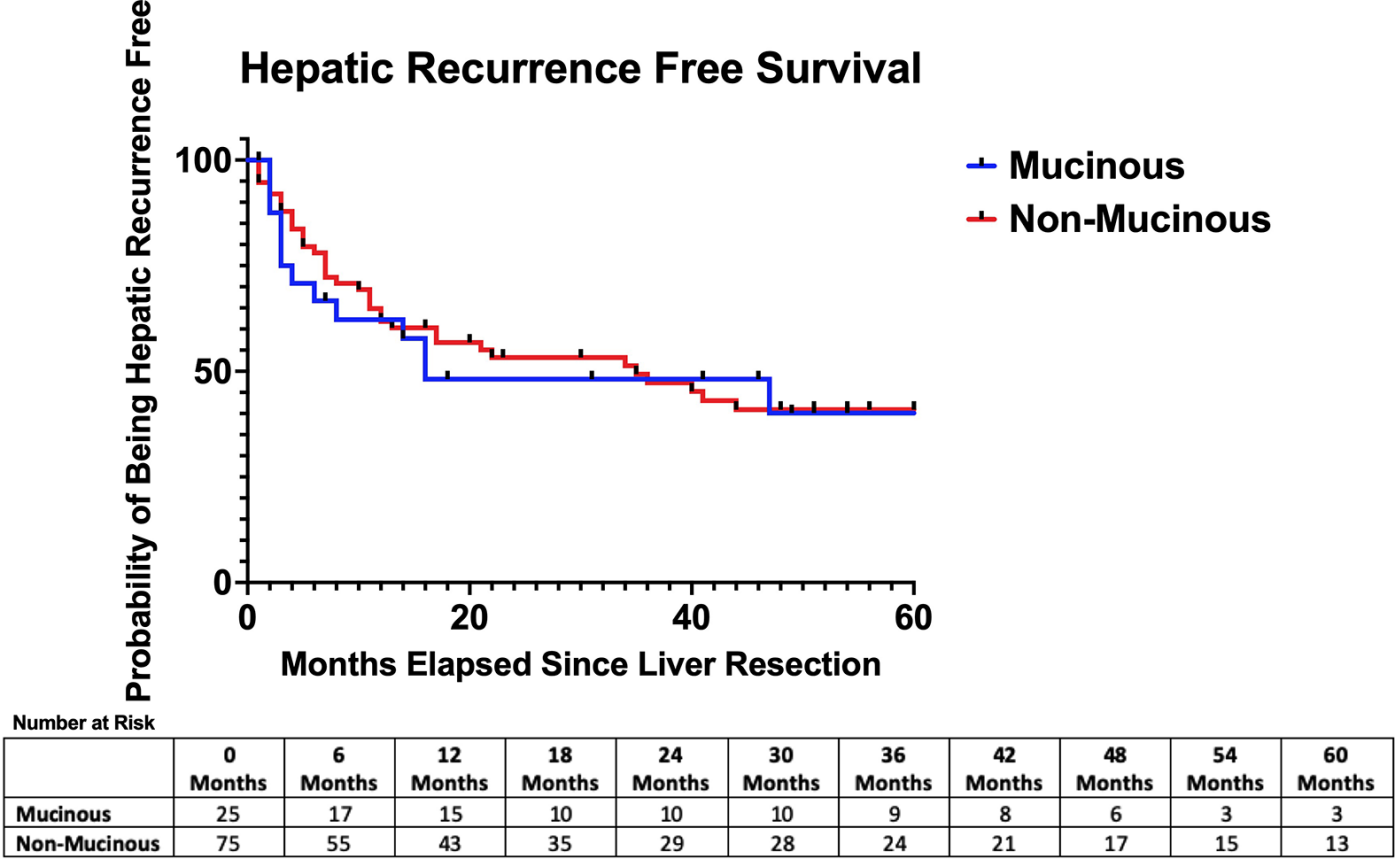
Hepatic recurrence-free survival after hepatic resection. Median hepatic recurrence free survival = 16 months (mucinous) versus 35 months (non-mucinous) *p* = 0.85.

**Figure 2:**
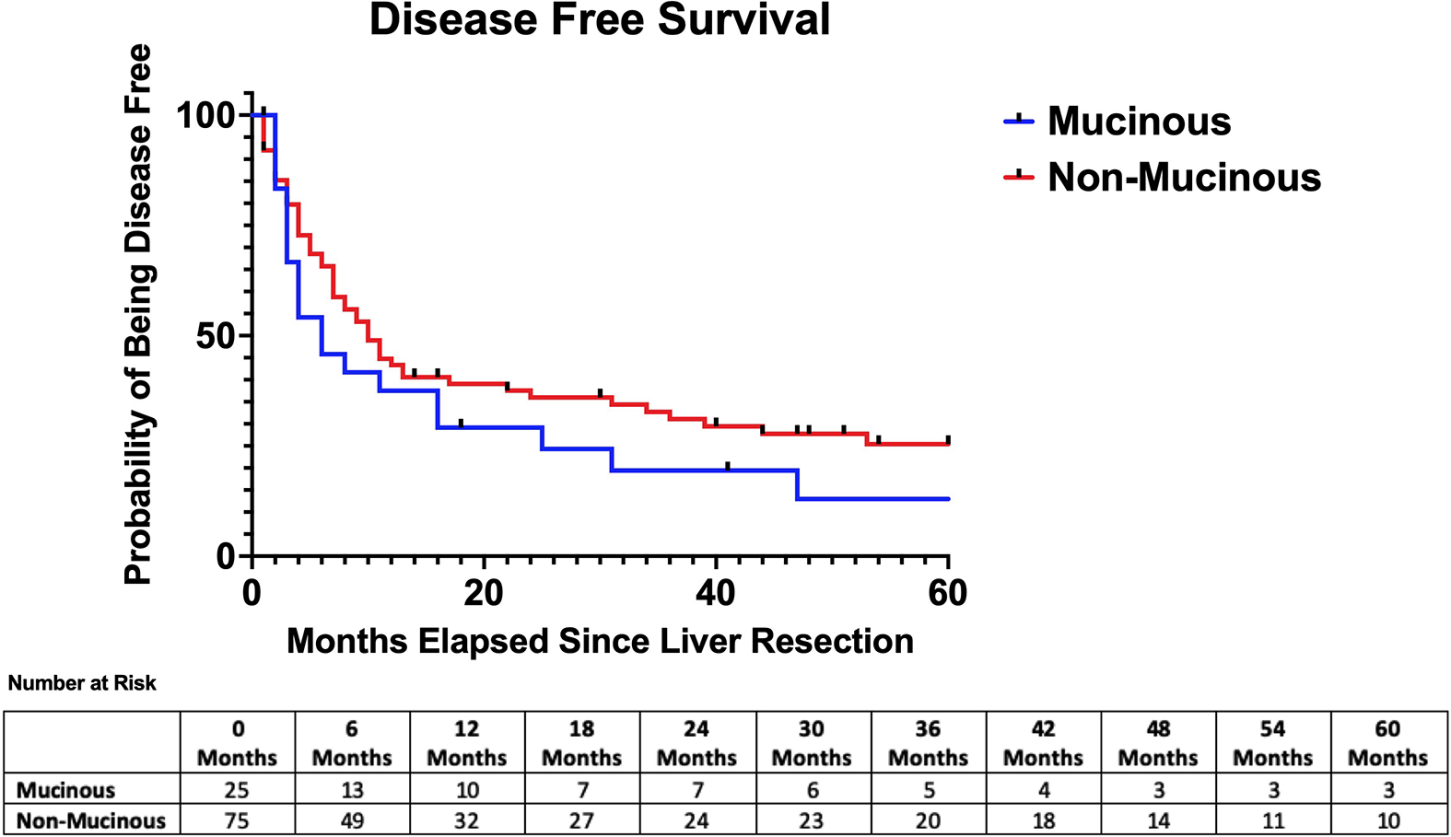
Disease-free survival after hepatic resection. Median disease free survival = 6 months (mucinous) versus 10 months (non-mucinous) *p* = 0.25.

**Figure 3:**
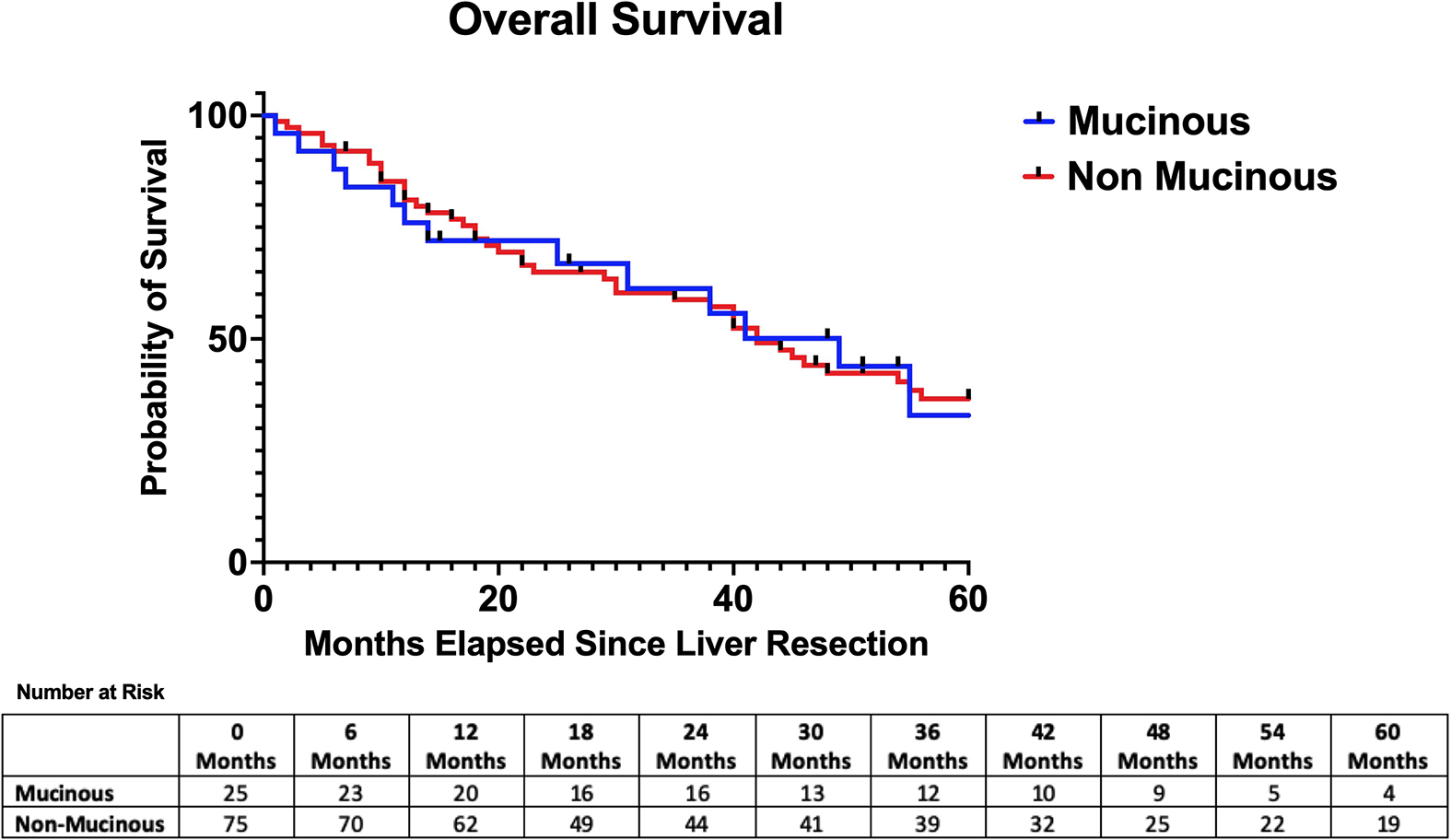
Overall survival after hepatic resection. Median overall survival = 49 months (mucinous) versus 42 months (non-mucinous) *p* = 0.98.

The median follow-up time was similar between the two groups (26 months vs 35 months, p=0.44). The median hepatic recurrence-free survival was 16 months in the mucinous group and 35 months in the adenocarcinoma NOS group (p=0.85) [See (**Figure 1**)]. The median disease-free survival was 6 months in the mucinous group and 10 months in the adenocarcinoma NOS group (p=0.25) [See (**Figure 2**)]. The median overall survival time was 49 months in the mucinous group and 42 months in the adenocarcinoma NOS group (p=0.98) [See (**Figure 3**)]. The 1, 3 and 5-year overall survival rates were 76%, 63% and 34% respectively in the mucinous group compared to 81%, 60% and 37% respectively in the adenocarinoma NOS group. Repeat hepatic resection for recurrence has not been statistically different between the two groups to date (24.0% vs 10.7%, p=0.11).

**Figure 4:**
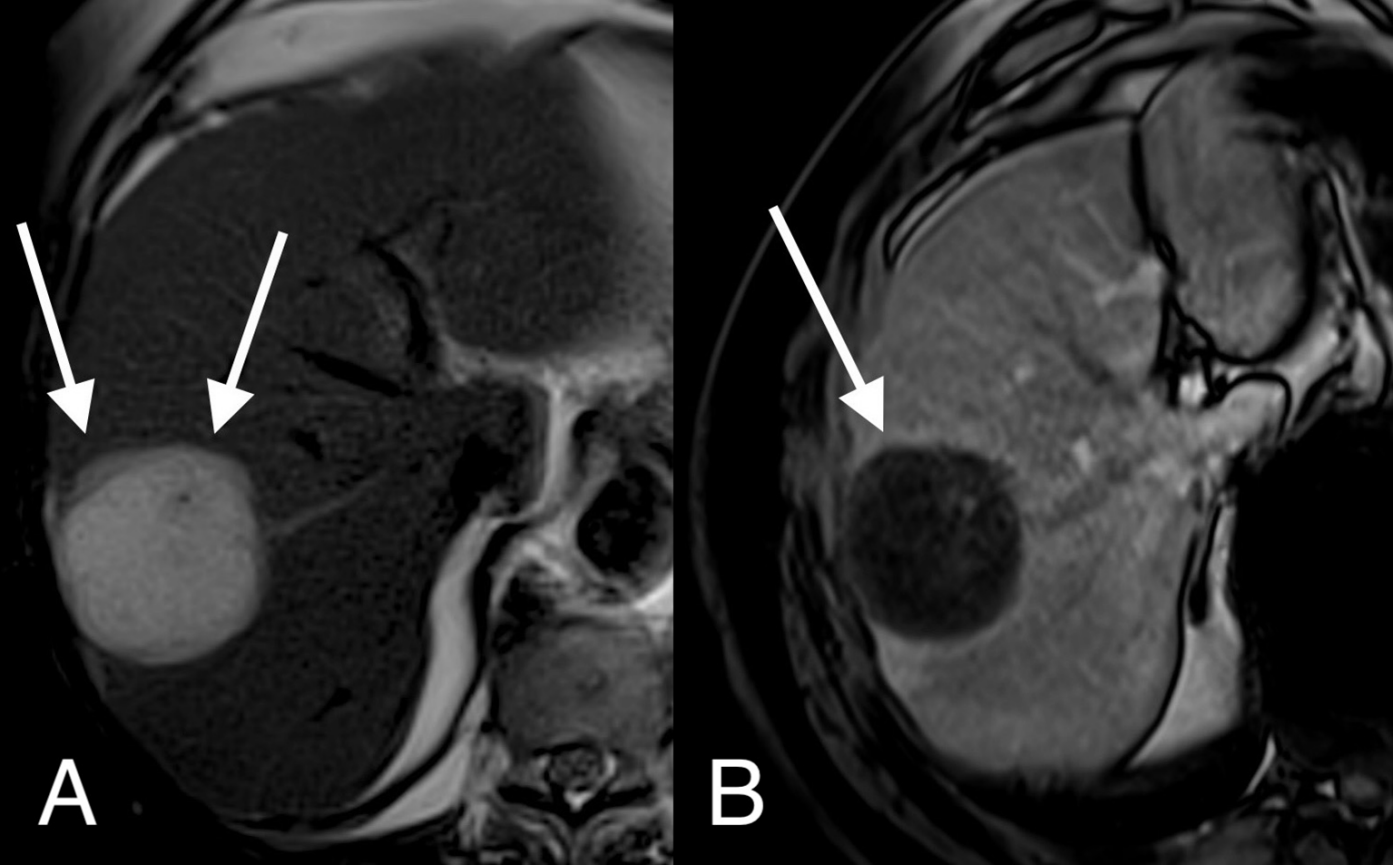
MRI image of a mucinous colorectal liver metastasis.Axial T2 weighted **(A)** and T1 weighted gadolinium enhanced **(B)** MRI images demonstrate a large T2 bright, cystic appearing lesion in segments 7 and 8 with a thick wall (arrows) and a thin rim of post-contrast enhancement.

In patients where preoperative MRI scans were available, a mucinous component was identified in the liver metastasis specimen of 75% of patients with liver metastases derived from mucinous primaries while the remaining 25% had solid metastases [See (**Figure 4A, B**)]. In patients with liver metastases derived from adenocarcinoma NOS primaries, 2.4% had a mucinous component in their liver metastasis, while the remaining 97.6% had solid metastases. MRI correctly idenitifed all the solid metastases in the mucinous group, however, it only identified the mucinous component in 26.7% of the mucin containing metastases. In the adenocarcinoma NOS group MRI identified 92.7% of patients with solid metastases correctly and the 2.4% of patients with metastases with a mucin component correctly. However, 4.9% of patients with solid tumours on histology were incorrectly identified as mucin containing tumours on MRI. In this series the sensitivity of preoperative MRI for detecting the presence of mucin in liver metastases was 31.3%, the specificity was 95.6%, the PPV was 71.4%, the NPV was 79.6% and the overall accuracy rate was 78.7%.

## Discussion

Our data has shown that mucinous CRCLM have similar clinicopathological features to CRCLM derived from adenocarcinoma NOS and can be resected with similar rates of liver-specific morbidity and minimal 30-day mortality. Furthermore, it appears that the median intervals to local and distant recurrence as well as overall survival is similar between the two groups. Based on this data we suggest that mucinous histology should not impact the decision to proceed with surgery in patients with potentially resectable isolated hepatic metastases.

Prior publications that demonstrate worse outcomes in patients with primary mucinous CRC and mucinous CRCLM should be interpreted with caution as they include a wider range of patients and in many cases the patients with mucinous CRCLM have adverse primary tumor pathology compared to patients with CRCLM from adenocarcinoma NOS (15, 19, 20, 26–28) [See (**Table 3**)]. Only a single previous publication has demonstrated a survival advantage for patients with mucinous CRCLM, however, this study included only 14 patients with mucinous CRCLM (16). In contrast to some of the previous publications, our two groups are matched for a range of clinicopathological variables and include only patients with isolated hepatic metastases at the time of surgery, however, the findings of previous studies with larger sample sizes showing worse outcomes in patients with mucinous CRCLM should still be acknowledged and respected. Despite the difference in histological appearance, these groups might be biologically quite similar, this is in part demonstrated by the similarities in the rates of mismatch repair deficiency (MMR), *RAS* and *BRAF* mutations seen between the groups. This is at odds with previous publications that have shown increased rates of MMR deficiency, *RAS* and *BRAF* mutations in mucinous tumours (29). We believe from our data that it may not be the histological subtype that determines outcome, rather it is more likely to be genotype, and tumours with an adverse genotype are probably more likely to develop multifocal unresectable metastatic disease. This could mean that mucinous tumours still retain a worse prognosis overall, however, a subset may have favorable molecular features that result in potentially resectable disease in the metastatic setting.

**Table 3:** Results of previously published papers comparing mucinous liver metastases to adenocarcinoma NOS liver metastases.

Authors	Country	Year of publication	Enrolment interval	Number with mucinous liver metastases	Number with non-mucinous liver metastases	Disease free survival	Overall Survival
Lupinacci et al	Brazil and France	2014	2000-2010	10	82	Not reported	Worse in mucinous
Huang et al	China	2020	2010-2013	306	5510	Worse in mucinous	Worse in mucinous
Li *et al*	China	2019	1999-2016	34	102	No difference	No difference
Viganò et al	Italy	2014	1998-2012	102	102	Worse in mucinous	Worse in mucinous
Bouviez et al	France	2014	1990-2000	14	72	Not reported	Improved in mucinous

The accuracy of preoperative MRI in determining the presence of mucin in a CRCLM appears to be limited based on the data available from our study, with a sensitivity of 31.3% when the radiologist is blinded to the histological subtype. While there are some publications documenting the features of mucinous CRCLM on different imaging modalities, there appears to be a paucity of data on the performance of MRI at detecting the presence of mucin in CRCLM (24, 30–32). There is a single study that demonstrates a sensitivity of 56% when a combination of MRI and PET are used to try and detect mucin in CRCLM, clearly the routine use of PET to determine the presence of mucin cannot be recommended with a sensitivity of only 56%. Interestingly, the same study also describes the sensitivity of MRI alone at detecting mucin in colorectal cancer metstases at all sites, and in keeping with our findings it is only 32% (21). The results from our study appear to represent the first time that data has been reported that correlates the histological findings with the MRI findings in the setting of mucinous CRCLM. Detecting the presence of mucin in CRCLM might be of academic interest, however, at present it is unlikely to influence any change in the management of the patient. While there is a theoretical argument that the presence of mucinous CRCLM might prompt the clinician to work harder to rule out occult peritoneal metastases prior to undertaking a liver resection, in most cases the histological subtype of CRC should already be available from the primary tumour (33). Although it appears that MRI lacks sensitivity for detecting mucin in CRCLM, the role of this imaging modality in determining the size, site, number, response to treatment and resectability of CRCLM is firmly established (34, 35).

There is limited data regarding the use of perioperative chemotherapy in the setting of mucinous CRCLM and the data that is available suggests that it does not provide a survival benefit to this cohort of patients (17). A lack of response to chemotherapy has also been noted with mucinous tumours in the primary setting (36–39). This data raises questions as to whether patients with mucinous CRCLM should be considered for up-front surgery or else offered alternative chemotherapeutic agents or targeted therapies. Answering these questions will prove difficult given the small numbers of mucinous tumours that are encountered and the even smaller number that go on to develop resectable isolated hepatic metastases. Clearly a multi-institutional collaborative approach will be required to learn more about this cohort. At the present time and until more data becomes available we recommend that patients with resectable isolated hepatic metastases derived from mucinous CRC are treated in a similar fashion to those with CRCLM derived from adenocarcinoma NOS. The timing and type of surgery as well as the use of perioperative chemotherapy and targeted therapy should be decided by a dedicated CRCLM multidisciplinary team. It is likely that genomic, transciptomic and proteomic data will be more widely used in the future to help guide treatment decisions and provide a more personalised approach to the perioperative treatment of CRCLM (40).

The main limitation of this study is the small number of patients with mucinous tumours that were included, this could potentially result in a type II error. The included patients represent all patients with mucinous CRCLM who have underwent surgery in our institution during the 12-year study period and the small number reflects the reality that this histological subtype of CRC is infrequently encountered, furthermore, identifying patients with isolated resectable hepatic metastases derived from mucinous CRC is an even less frequently encountered event. Another limitation is the lack of data available on targeted therapy and specific perioperative systemic therapy regimens, unfortunately this data is not available as the majority of patients included in the study are referred to our institution from a number of external centres for the surgical management of their disease, while the oncological aspect of their treatment is carried out in the referring insitution. The proportion of mucin to tumor in the liver metastases was not available, this may be relevant when determining the sensitivity of MRI at detecting mucin in liver lesions, for example MRI might be more sensitive when the percentage of mucin in the lesion is greater than 50%. We are currently in the process of documenting the percentage of mucin as well as several other advanced pathological markers in this cohort as part of another ongoing project, the data of which will hopefully be available in the near future. Finally, the follow-up interval for some of the more recently operated on patients is short. The study does however consist of extensively matched groups with complete clinical, pathological, molecular and follow up data available for each case.

## Conclusion

Based on our single institution data, resection of CRCLM derived from mucinous adenocarcinoma appears to be feasible, safe and associated with similar oncological outcomes to that of adenocarcinoma NOS. At present there is no strong evidence to suggest that the histological subtype of CRCLM should have any impact on the decision making process regarding surgical resection for patients with metastatic CRC. Multimodal treatment options for patients with mucinous CRCLM should be decided by a dedicated hepatobiliary multidisciplinary team. Recent advances in systemic treatment such as targeted therapy and novel surgical strategies including staged procedures has helped to improve outcomes for patients regardless of histological subtype. Despite the fact that MRI lacks sensitivity in detecting mucin in the setting of CRCLM, this imaging modality retains an important place in the preoperative work up of patients with CRCLM.

## Data Availability Statement

The raw data supporting the conclusions of this article will be made available by the authors, without undue reservation.

## Ethics Statement

The studies involving human participants were reviewed and approved by SVUH Audit Committee. Written informed consent for participation was not required for this study in accordance with the national legislation and the institutional requirements.

## Author Contributions

Study concept and design – All authors. Study materials – All authors. Data collection – IR. Statistical analysis – IR and ER. Manuscript preparation – All authors. Manuscript review – all authors. All authors contributed to the article and approved the submitted version.

## Conflict of Interest

The authors declare that the research was conducted in the absence of any commercial or financial relationships that could be construed as a potential conflict of interest.

## Publisher’s Note

All claims expressed in this article are solely those of the authors and do not necessarily represent those of their affiliated organizations, or those of the publisher, the editors and the reviewers. Any product that may be evaluated in this article, or claim that may be made by its manufacturer, is not guaranteed or endorsed by the publisher.
